# Supplementary material for: Low winter precipitation, but not warm autumns and springs, threatens mountain butterflies in middle-high mountains
Source: PeerJ. 2021 Aug 27;9:e12021. doi: 10.7717/peerj.12021 (PMC8404571; doi:10.7717/peerj.12021)
Supplement: Supplemental Information 1 — We provide more detailed information on the methods of butterfly monitoring and additional results on the population recruitment curves, correlations among climate variables, temporal trends in the data, and the effects of climate variables on the abundance and phenology of Erebia epiphron and E. sudetica (Figs. S1–S19 and Tables S2–S4). This file contains Figs. S1–S19 and Tables S2–S4. [file peerj-09-12021-s001.pdf]

Low winter precipitation, but not warm autumns and springs, threatens mountain butterflies in middle-high mountains

Martin Konvicka[1,2], Tomas Kuras[3], Jana Liparova[2], Vit Slezak[4], Dita Horazna[2], Jan Klecka[2], Irena Kleckova[2*]

[1] Faculty of Science, University of South Bohemia, Branišovská 31, CZ-37005 České Budějovice, Czech Republic
[2] Czech Academy of Sciences, Biology Centre, Institute of Entomology, Branišovská 31, CZ-37005 České Budějovice, Czech Republic
[3] Faculty of Science, Palacký University Olomouc, 17. Listopad 1192/12, CZ-77146 Olomouc, Czech Republic
[4] Jeseníky PLA Administration, Šumperská 93, CZ-79001 Jeseník, Czech Republic

correspondence: irena.slamova@gmail.com

Supporting Information

We provide more detailed information on the methods of butterfly monitoring and additional results on the population recruitment curves, correlations among climate variables, temporal trends in the data, and the effects of climate variables on the abundance and phenology of *Erebia epiphron* and *E. sudetica* (Figures S1–S19 and Tables S2–S4). This file contains Figures S1–S19 and Tables S2–S4, while Table S1 is provided as a separate file. Table S1 summarises our data on the abundance of the two *Erebia* species and available climate variables.

Supplementary methods

Monitoring of adult abundances in the Jeseník Mts (J) and Krkonoše Mts (K) was done by counting butterflies along three transects in J and four transects in K. J1 (length 2800 m, altitude 1325–1465 m, centre coordinates N 50°2.948', E 17°13.359') follows the main ridge covered by *Nardus* grasslands; J2 (570 m, 1370–1400 m, N 50°3.874', E 17°14.678') crosses, in addition to *Nardus* grasslands, some *Pinus mugo* shrubs; and J3 (800 m, 1330–1492 m, N 50°4.770', E 17°13.880') ascends perpendicularly to the highest summit, from the tall-herb timber belt formations to *Nardus* grasslands. K1 (960 m, 1120–1270 m, N 50°41'27.5" E 15°38'39.5") and K2 (2400 m, 1270–1340 m, N 50°42'05.2" E 15°39'52.8") are located at cultural grasslands in the mountain zone; whereas K3 (1200 m, 1340–1360 m, N 50°42'42.3" E 15°40'33.5") and K4 (2500 m, 1360–1510 m, N 50°43'19.2" E 15°41'14.6") cross the subalpine habitats.

Table S1. (provided as a separate file) Summary data on the abundance monitoring and the climate variables are included: Walks = the number of transect walks completed during the year, Individuals_recorded = the total number of individuals observed during the year at the transect, Interval = the dates of the start and end of the field sampling. The fit of the generalised additive models (GAM) fitted for data from each transect and year separately is described: GAM_explained_dev = % deviance explained, GAM_df = the number of degrees of freedom

describing the complexity of the fitted curve, GAM_F = the F statistic value, GAM_P = the P-value. The fitted GAM was used to estimate the abundance index and descriptors of phenology: *PAI* = the population abundance index, *Onset* = the estimated onset of the flight period, *Duration* = the estimated duration of the flight period. The rest of the variables describe the climate fluctuations based on data from the available weather stations. See the Methods in the main text for detailed explanation of the variables.

Population recruitment curves

We monitored adults of *Erebia sudetica* and *Erebia epiphron* along three permanent transects in the Jeseník Mts (J1–J3) and four transects in the Krkonoše Mts (K1–K4) (see Figure 1 in the main text). Figures S1–S11 show the daily numbers of individuals of the two *Erebia* species in individual transects, standardised to account for differences in transect lengths, together with curves fitted by generalised additive models (GAM) separately for each transect and year.

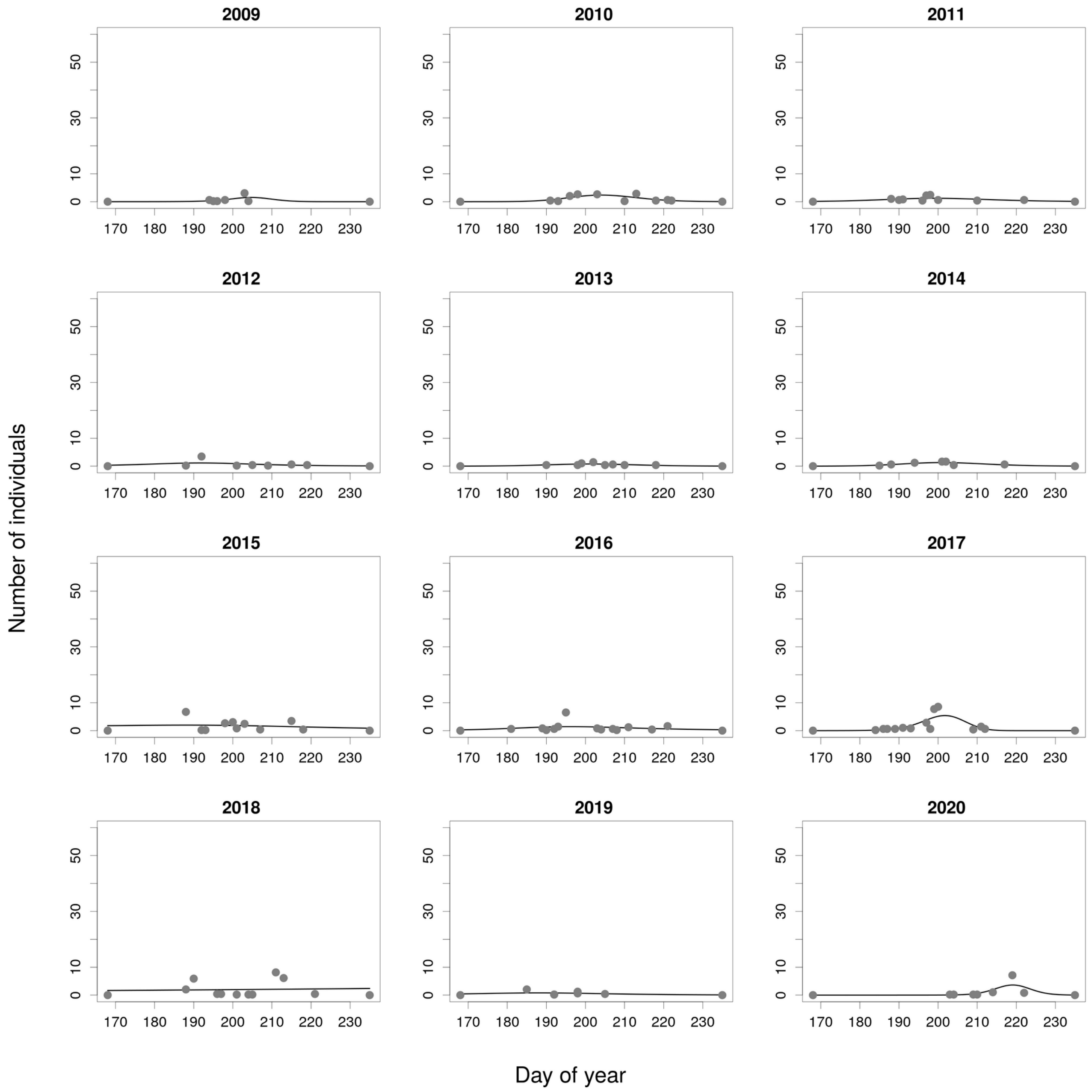

Fig. S1. *Erebia sudetica*, Jeseník Mts, transect J1. The curves were fitted by generalised additive models (GAM).

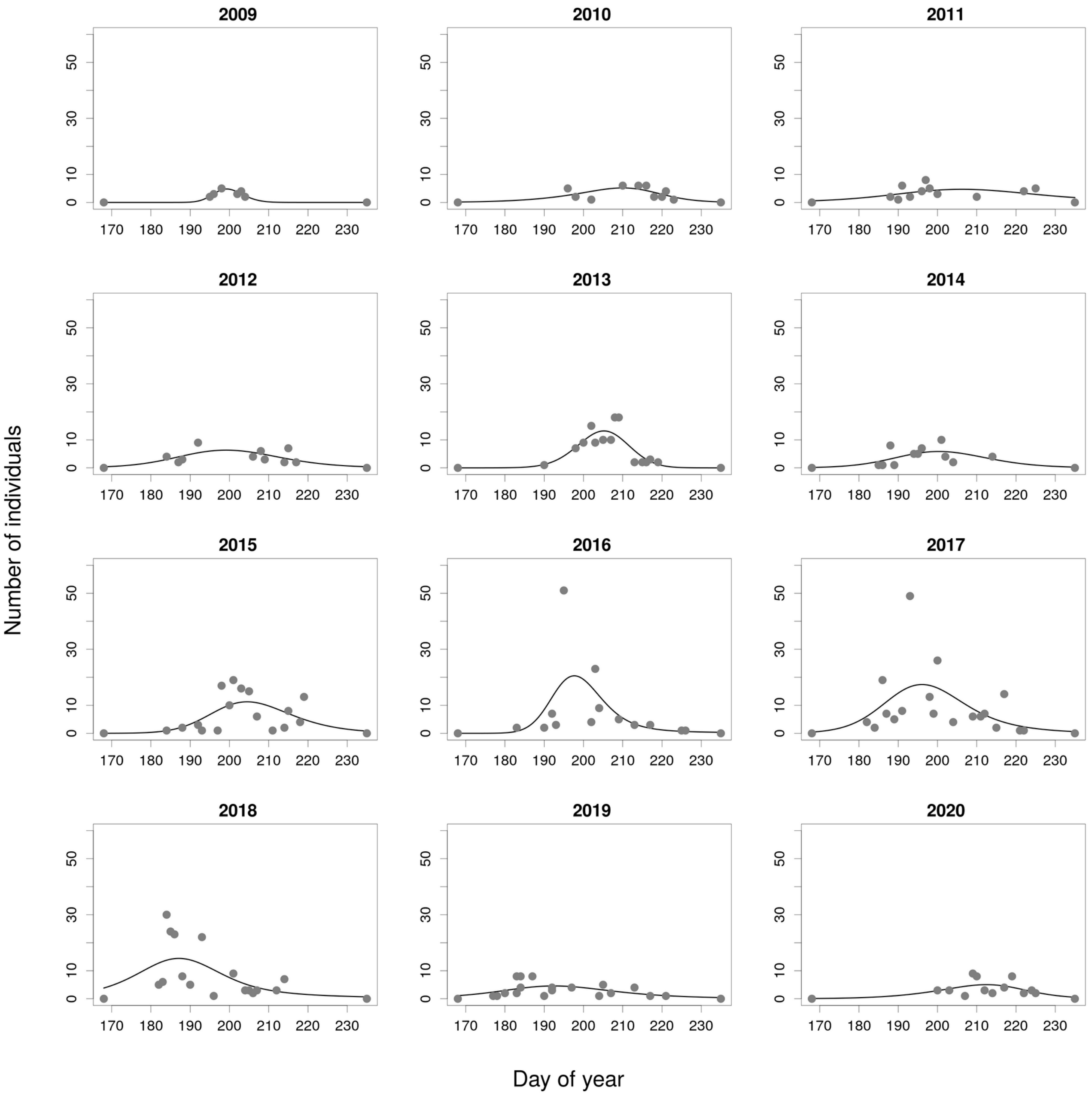

Fig. S2. *Erebia sudetica*, Jeseník Mts, transect J2. The curves were fitted by generalised additive models (GAM).

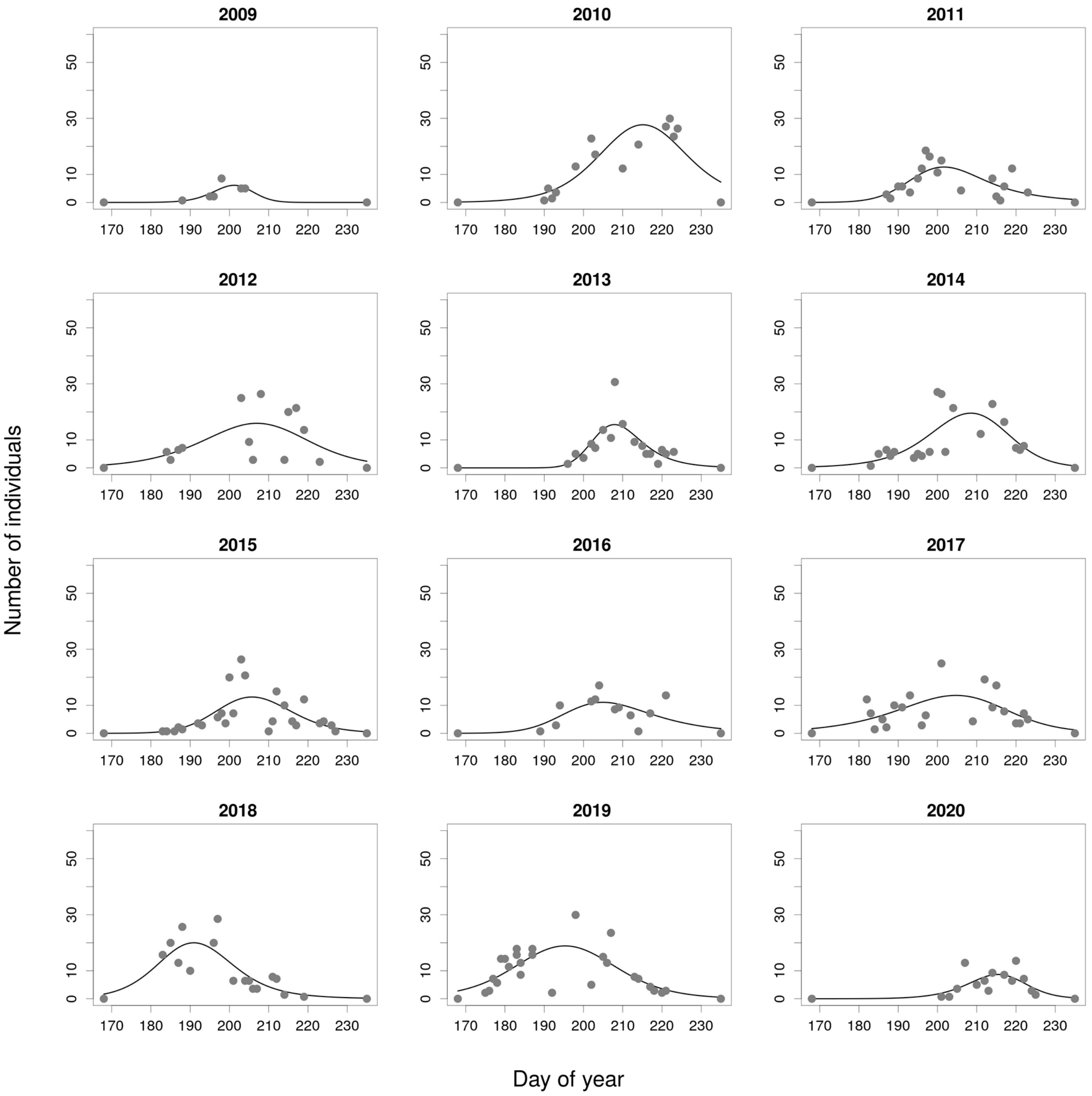

Fig. S3. *Erebia sudetica*, Jeseník Mts, transect J3. The curves were fitted by generalised additive models (GAM).

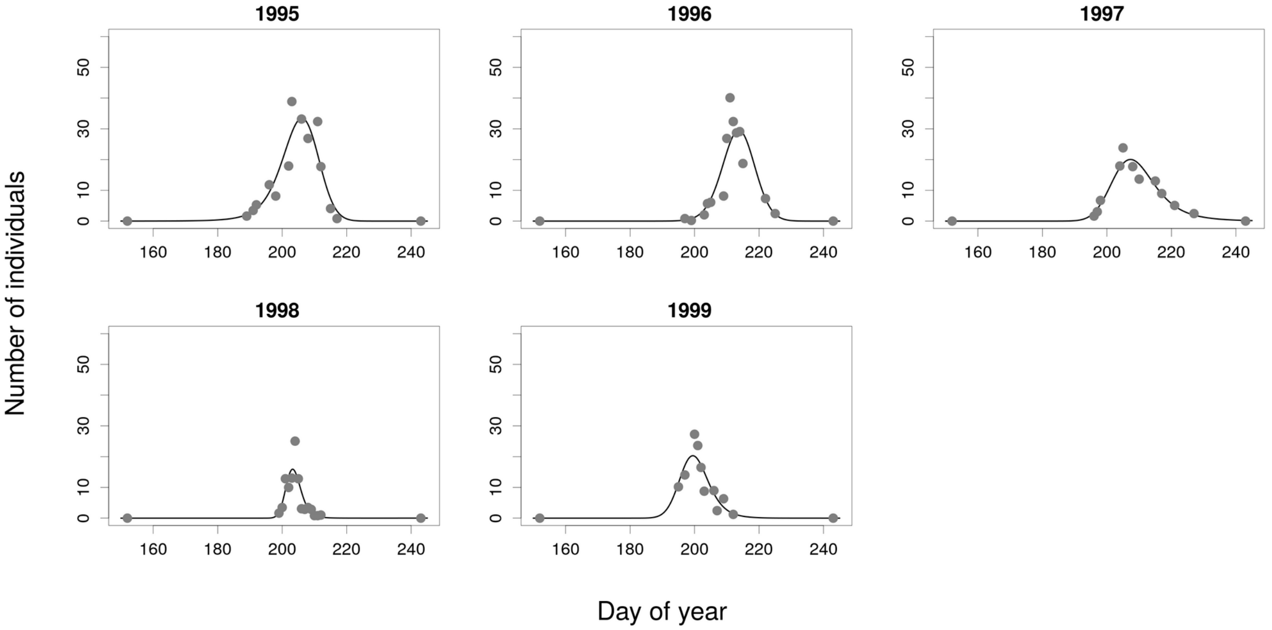

Fig. S4. *Erebia epiphron*, Jeseník Mts, transect J1, years 1995–1999. The curves were fitted by generalised additive models (GAM).

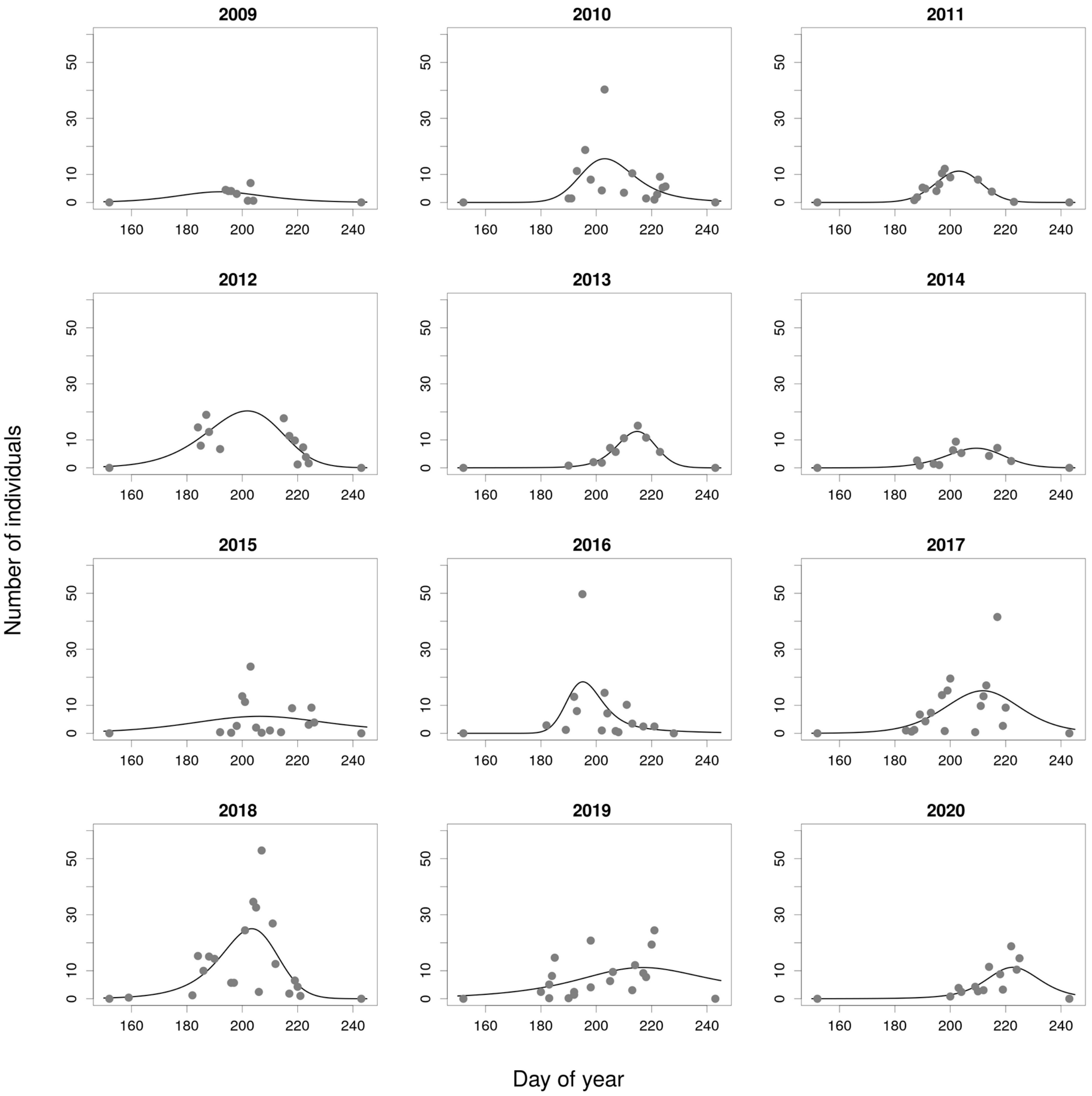

Fig. S5. *Erebia epiphron*, Jeseník Mts, transect J1, years 2009–2020. The curves were fitted by generalised additive models (GAM).

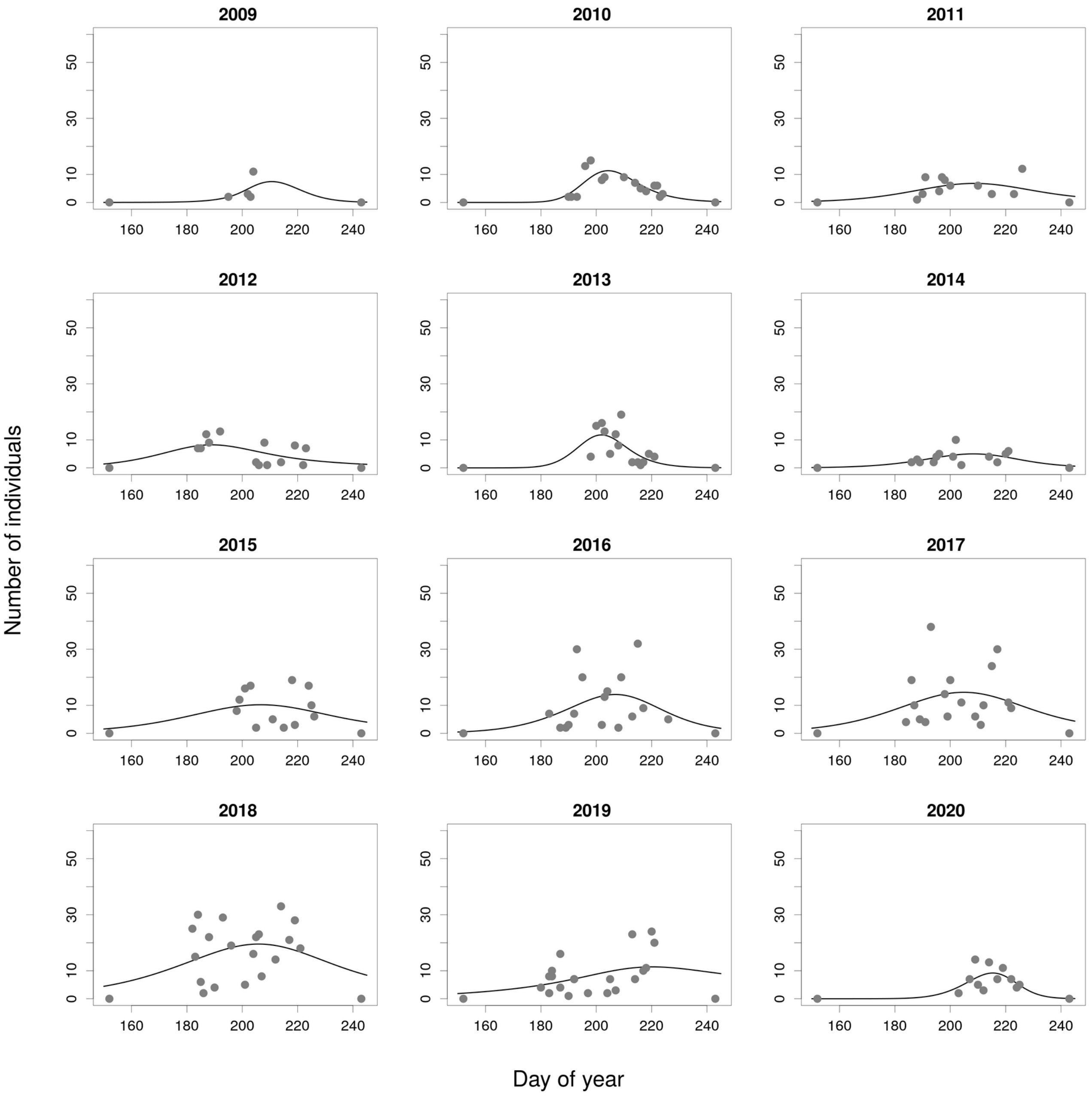

Fig. S6. *Erebia epiphron*, Jeseník Mts, transect J2, years 2009–2020. The curves were fitted by generalised additive models (GAM).

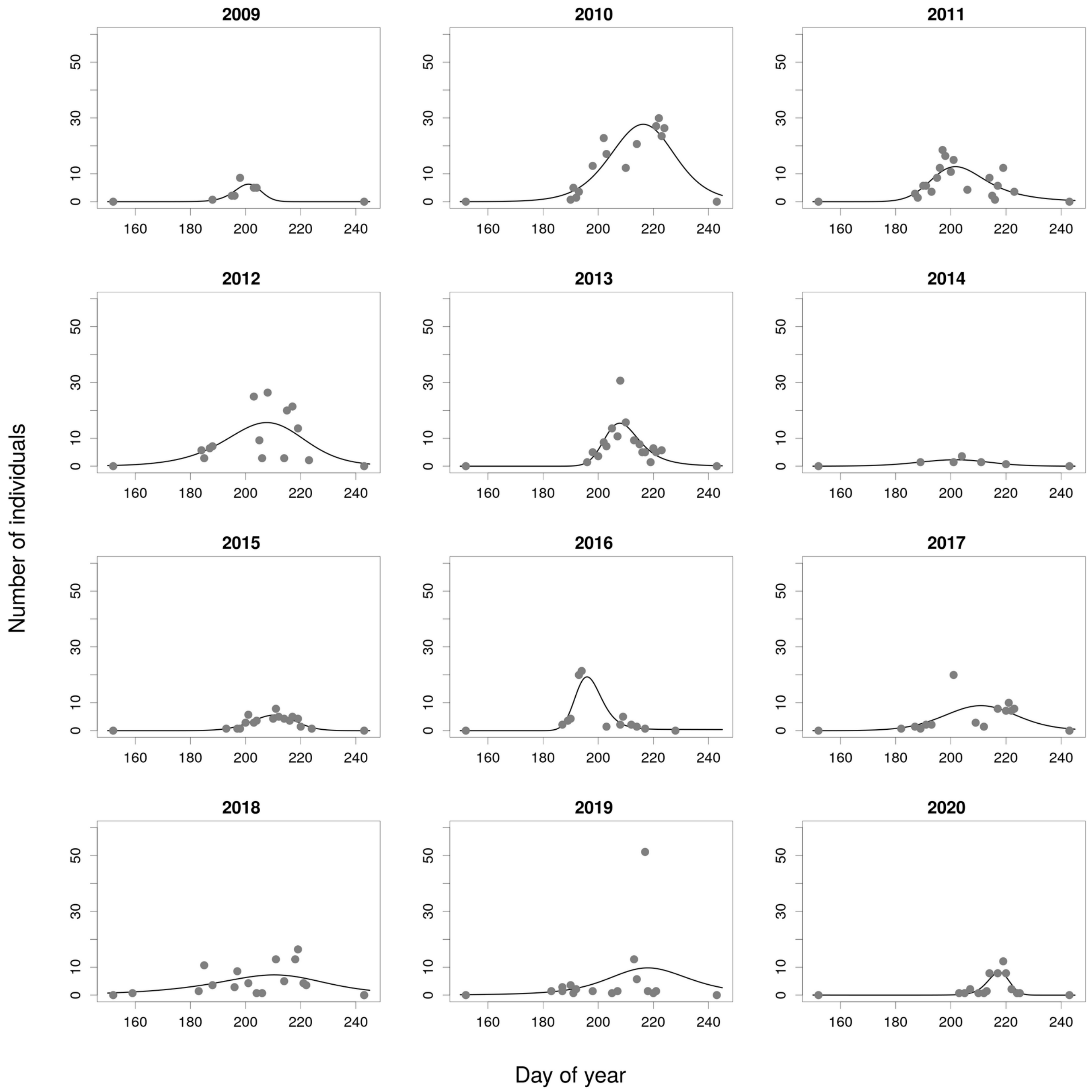

Fig. S7. *Erebia epiphron*, Jeseník Mts, transect J3, years 2009–2020. The curves were fitted by generalised additive models (GAM).

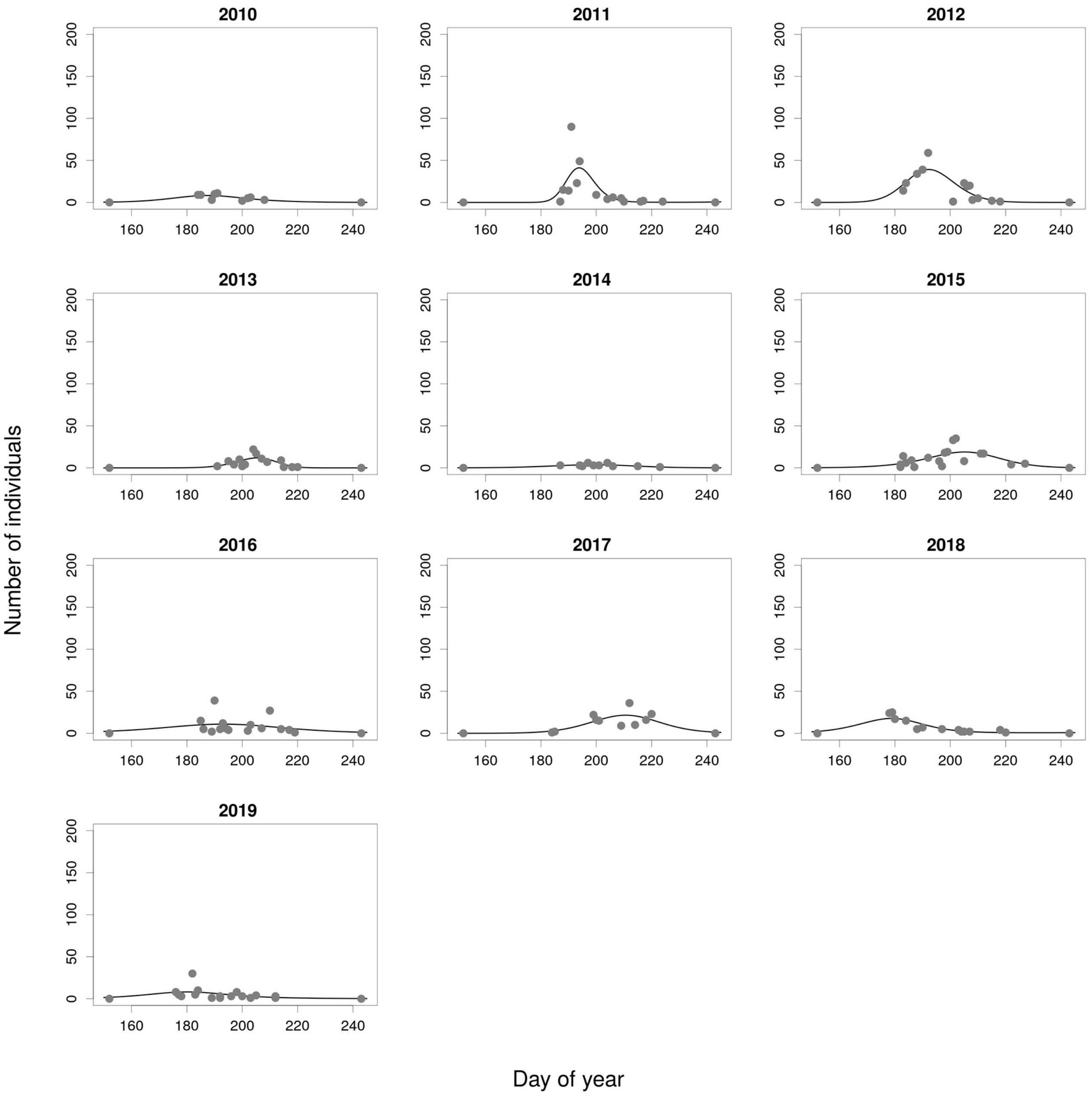

Fig. S8. *Erebia epiphron*, Krkonoše Mts, transect K1. The curves were fitted by generalised additive models (GAM).

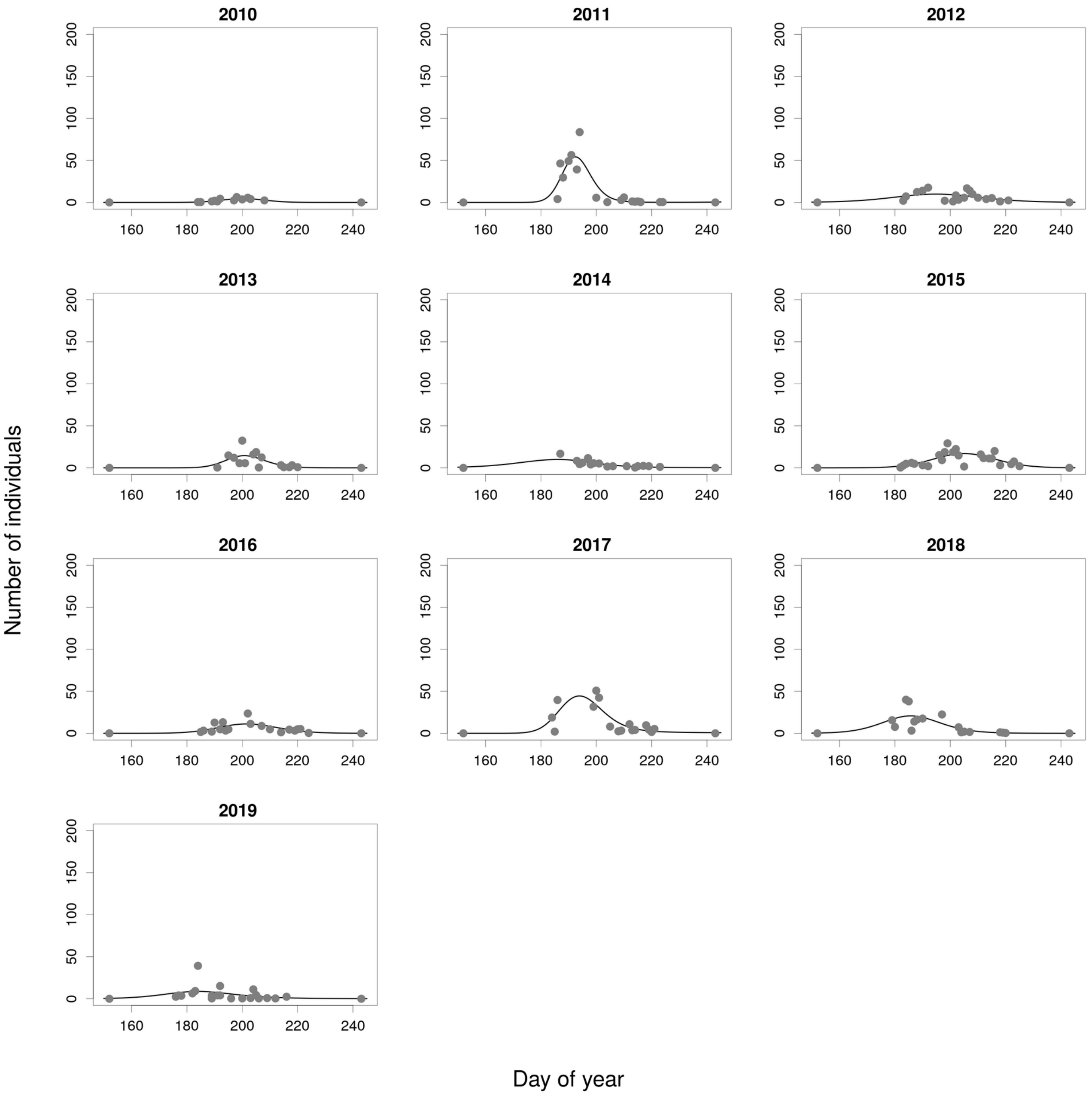

Fig. S9. *Erebia epiphron*, Krkonoše Mts, transect K2. The curves were fitted by generalised additive models (GAM).

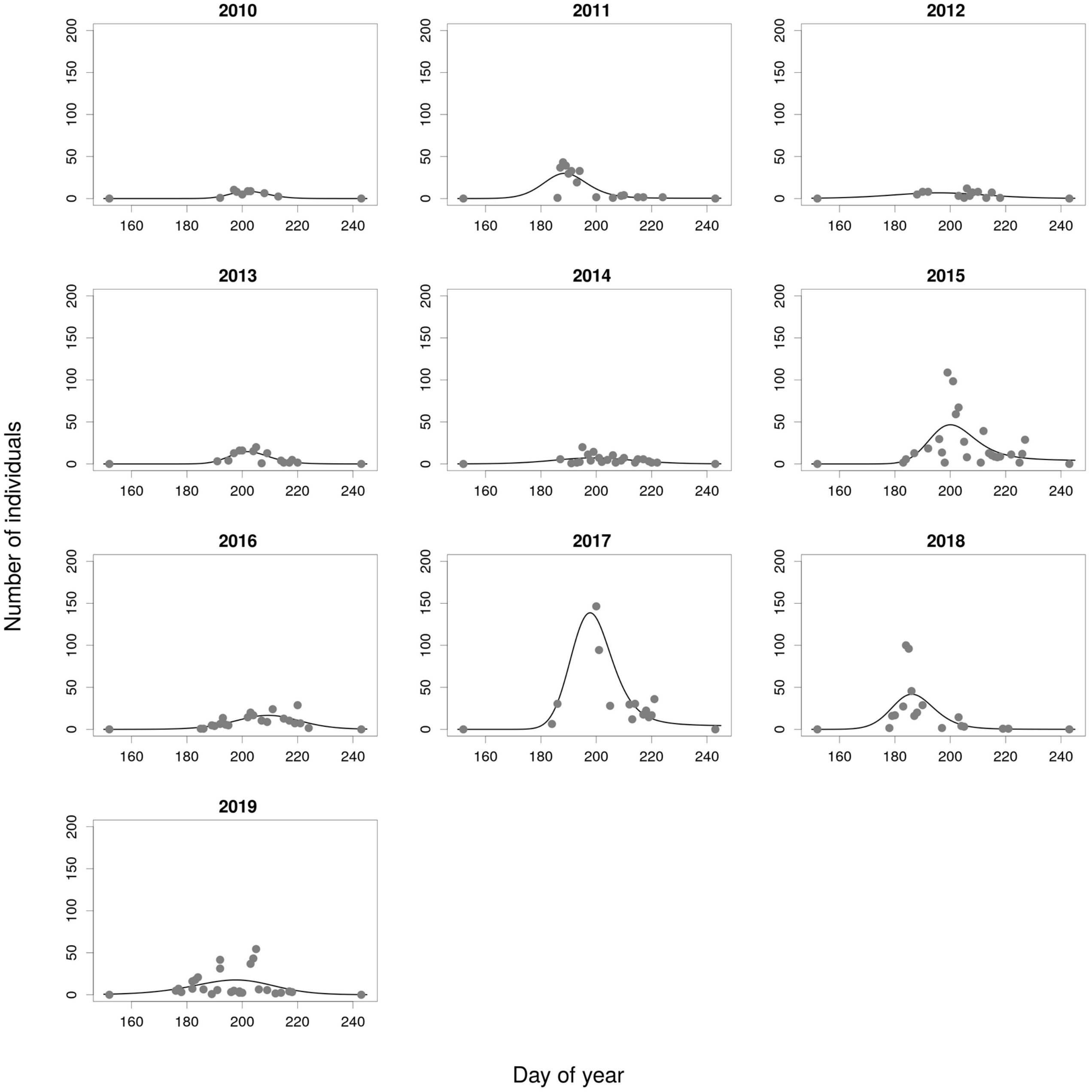

Fig. S10. *Erebia epiphron*, Krkonoše Mts, transect K3. The curves were fitted by generalised additive models (GAM).

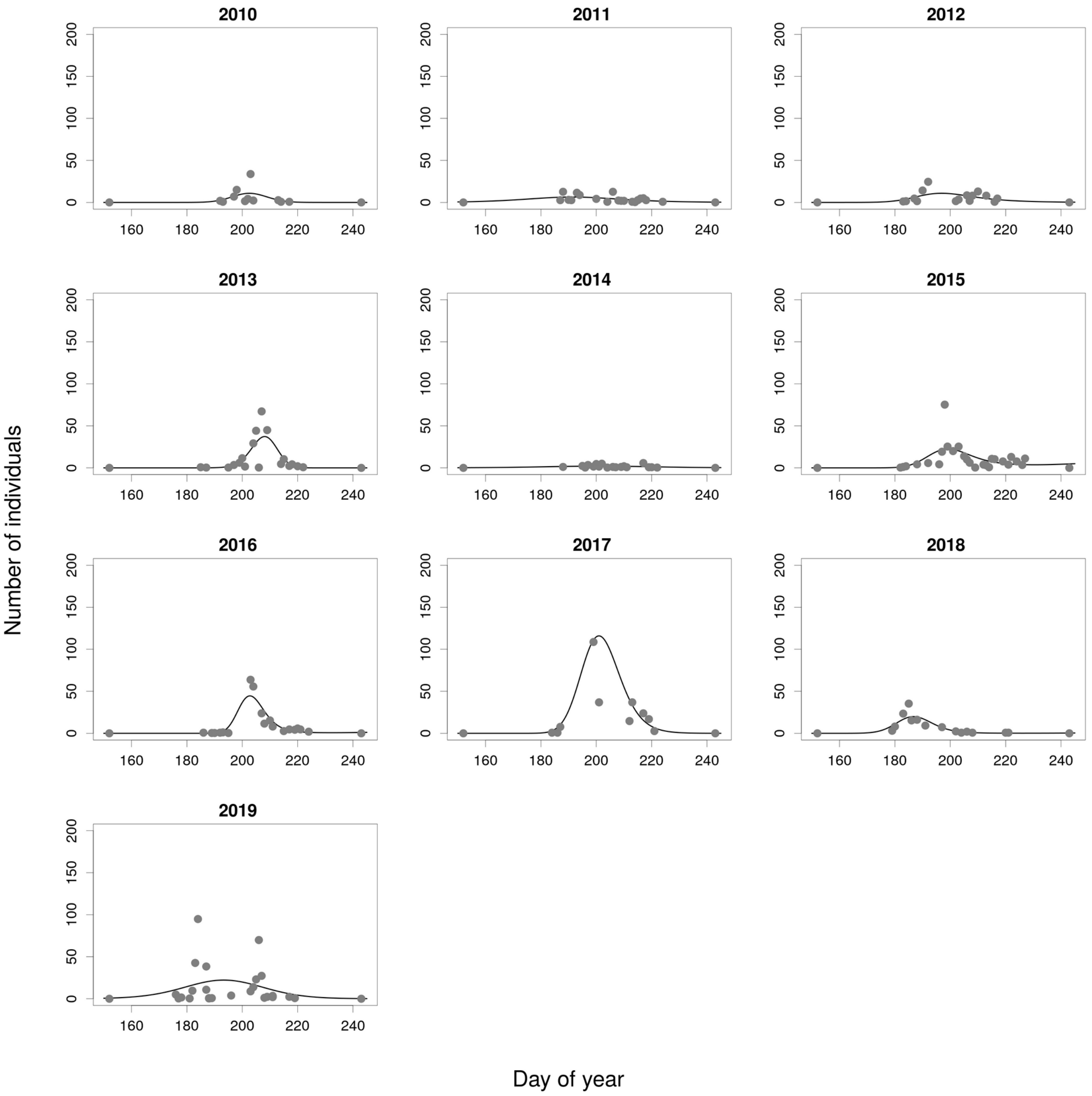

Fig. S11. *Erebia epiphron*, Krkonoše Mts, transect K4. The curves were fitted by generalised additive models (GAM).

Correlations among climate variables

Figures S12–S15 show Pearson’s correlation coefficients among all pairs of climate variables calculated separately for the Jeseník Mts (Fig. S12 and S13) and the Krkonoše Mts, where we used data from two weather stations – one at a lower altitude (Fig. S14) and another at a higher altitude (Fig. S15). In addition, we visualised the relationships among the climate variables also using PCA, with the variables scaled to unit variance (Fig. S16). The explanation of the variables is provided in the Methods in the main text.

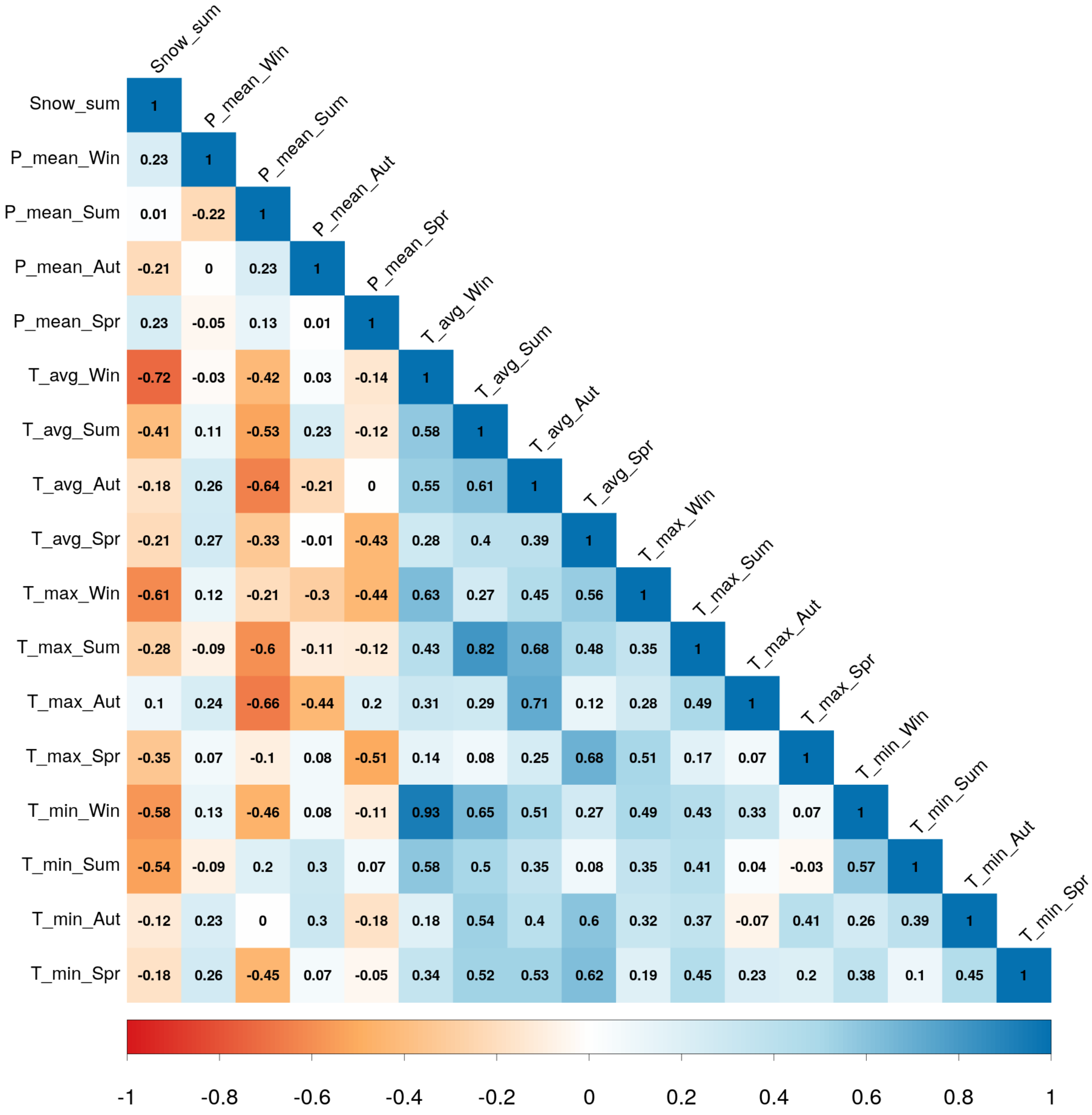

Fig. S12. Matrix of Pearson’s correlation coefficients among climate variables in the Jeseník Mts; complete data from years 1995–1997 and 2009–2020.

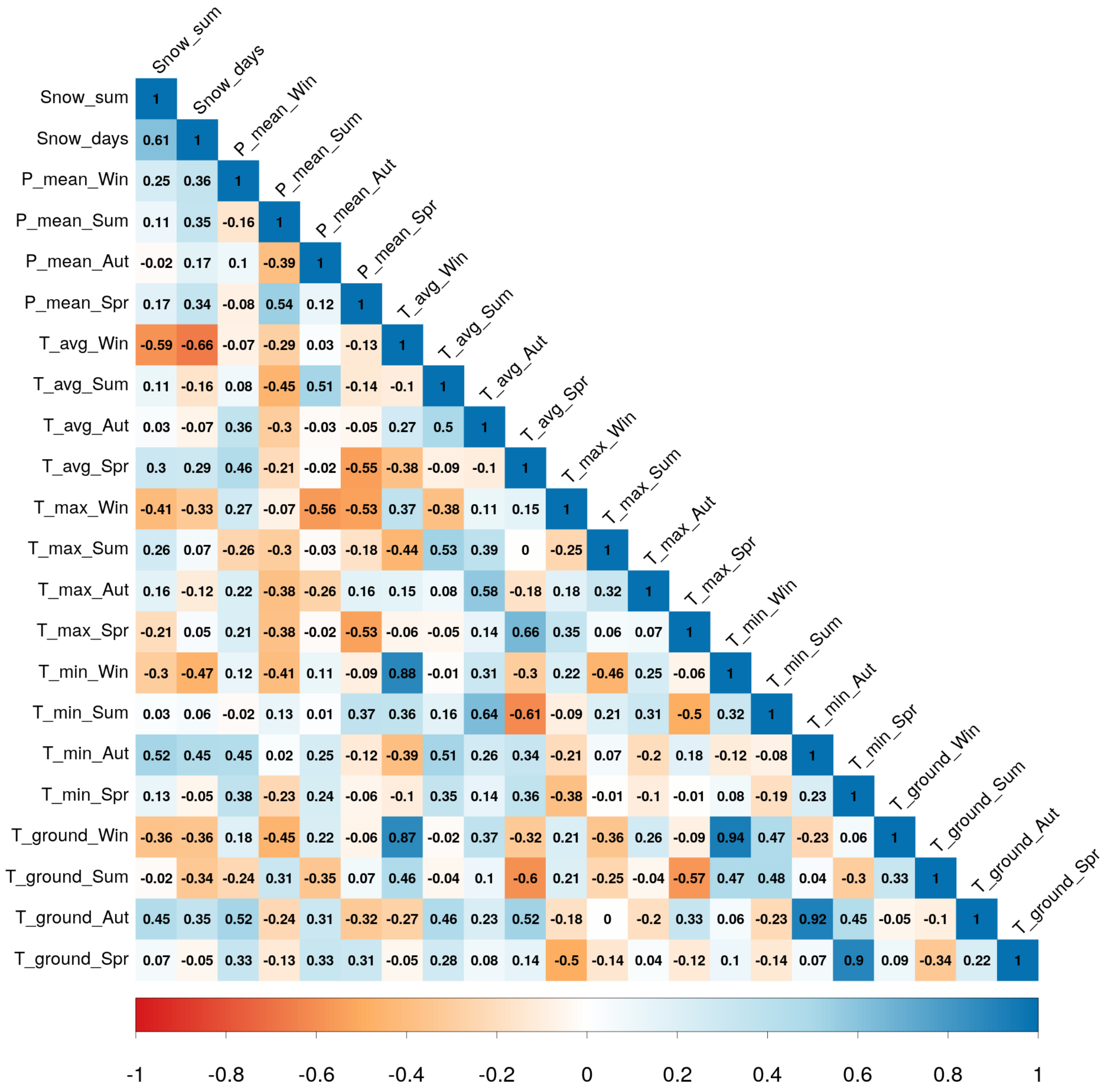

Fig. S13. Matrix of Pearson's correlation coefficients among climate variables in the Jeseník Mts; recent years only (2009–2020).

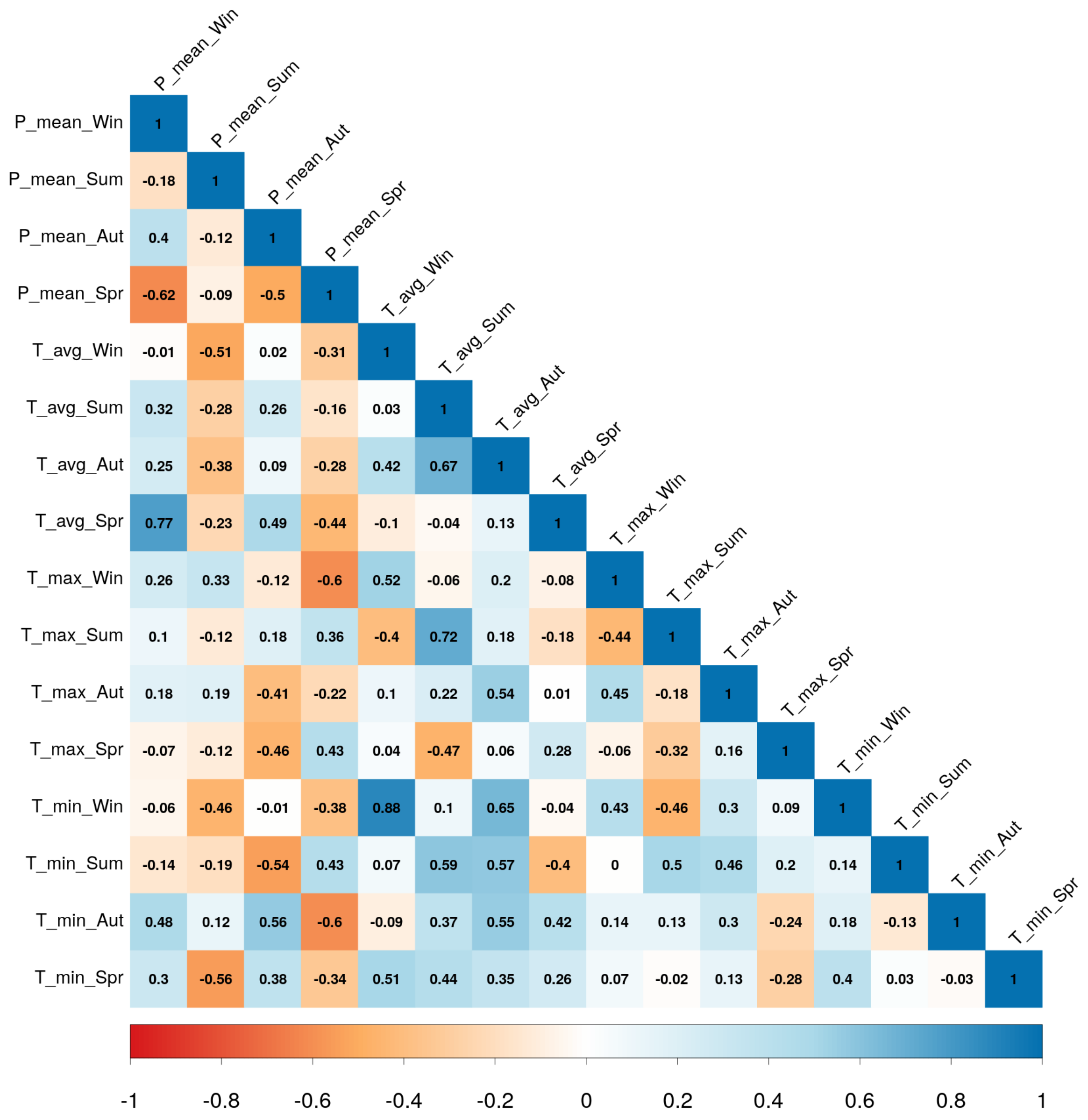

Fig. S14. Matrix of Pearson's correlation coefficients among climate variables in the Krkonoše Mts; data from years 2010–2019, weather station near transects K1 and K2.

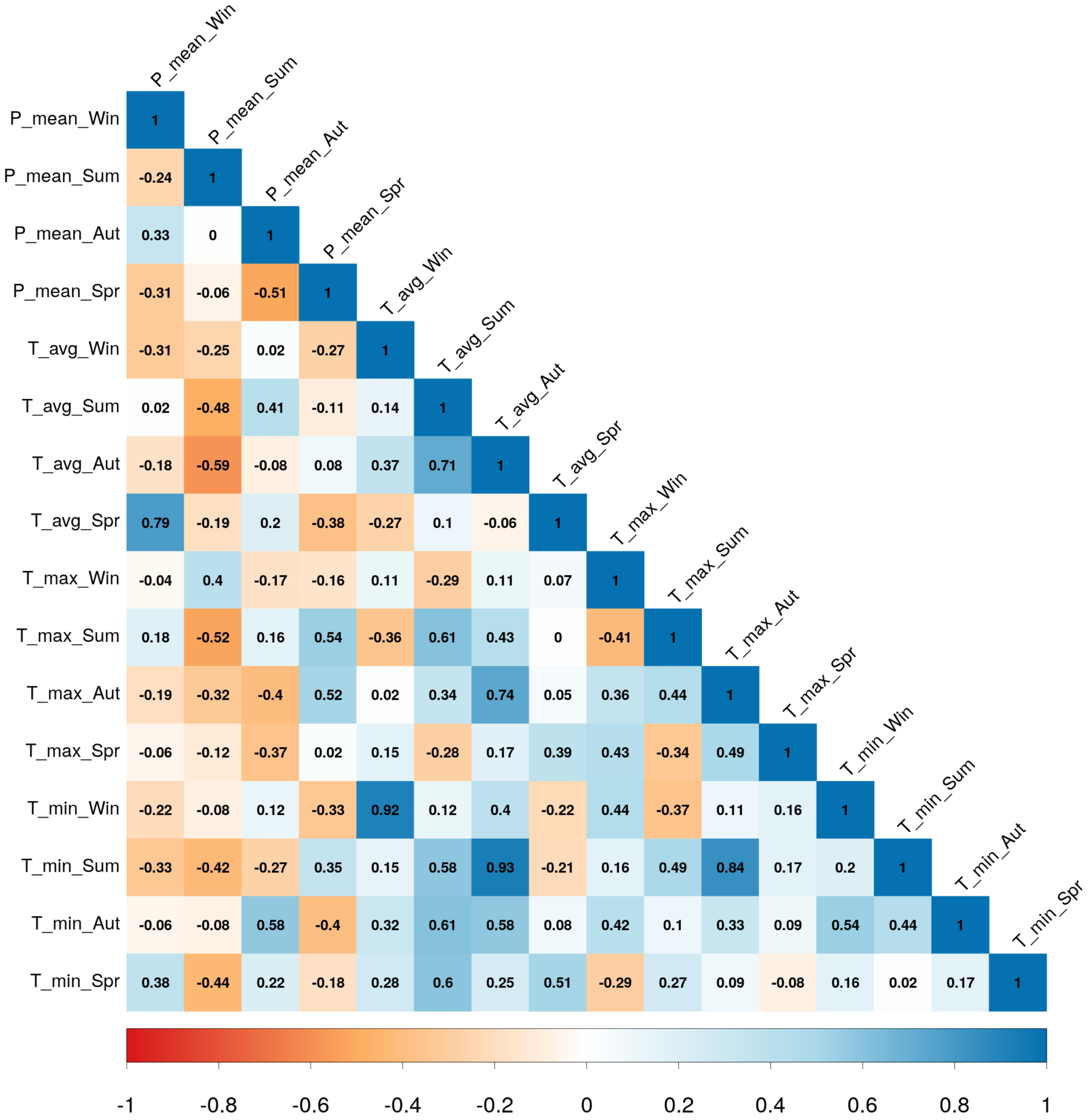

Fig. S15. Matrix of Pearson's correlation coefficients among climate variables in the Krkonoše Mts; data from years 2010–2019, weather station near transects K3 and K4.

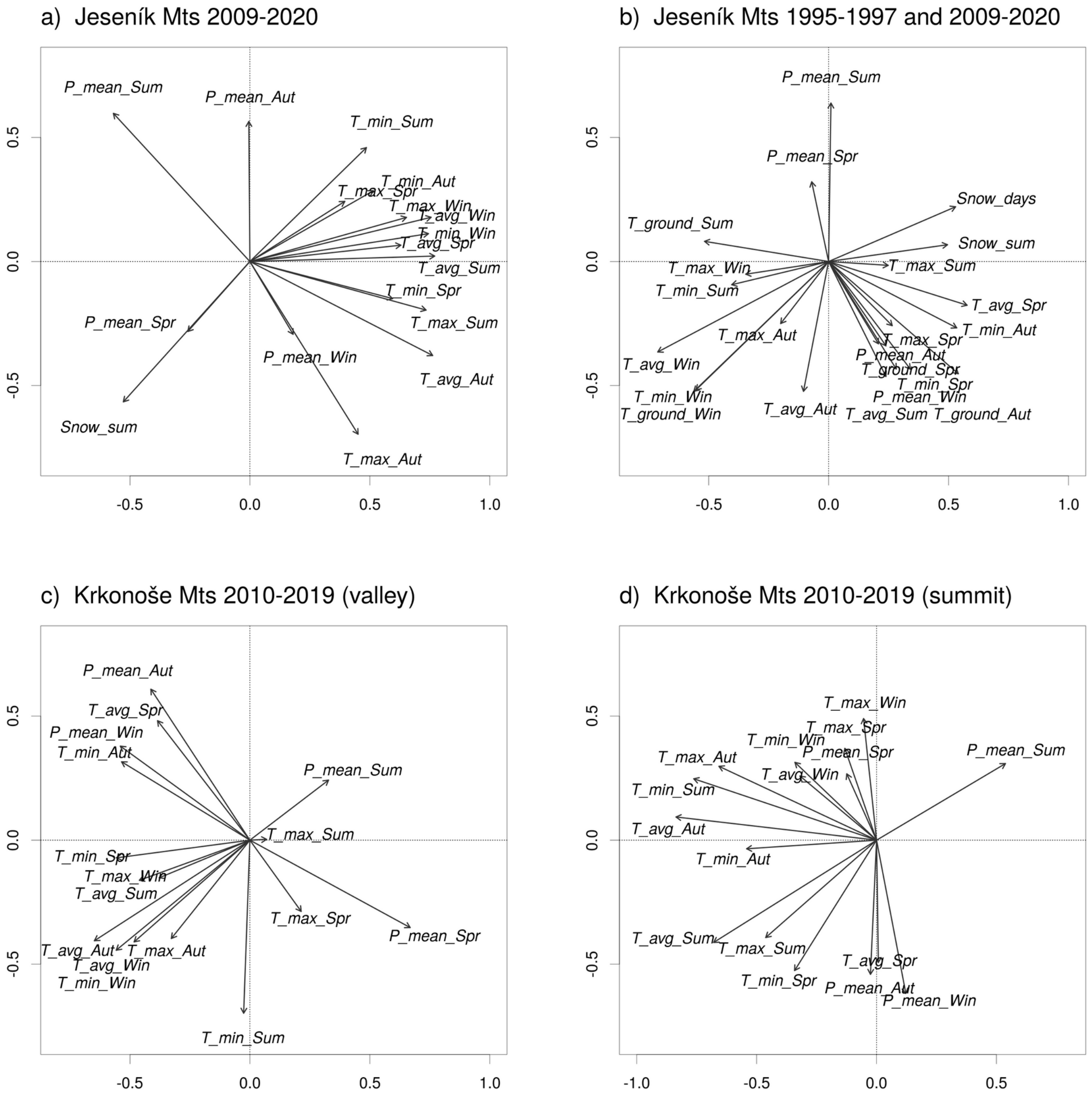

Fig. S16. PCA of the climate variables. The first two PCA axes are displayed.

The relationship between abundance and phenology

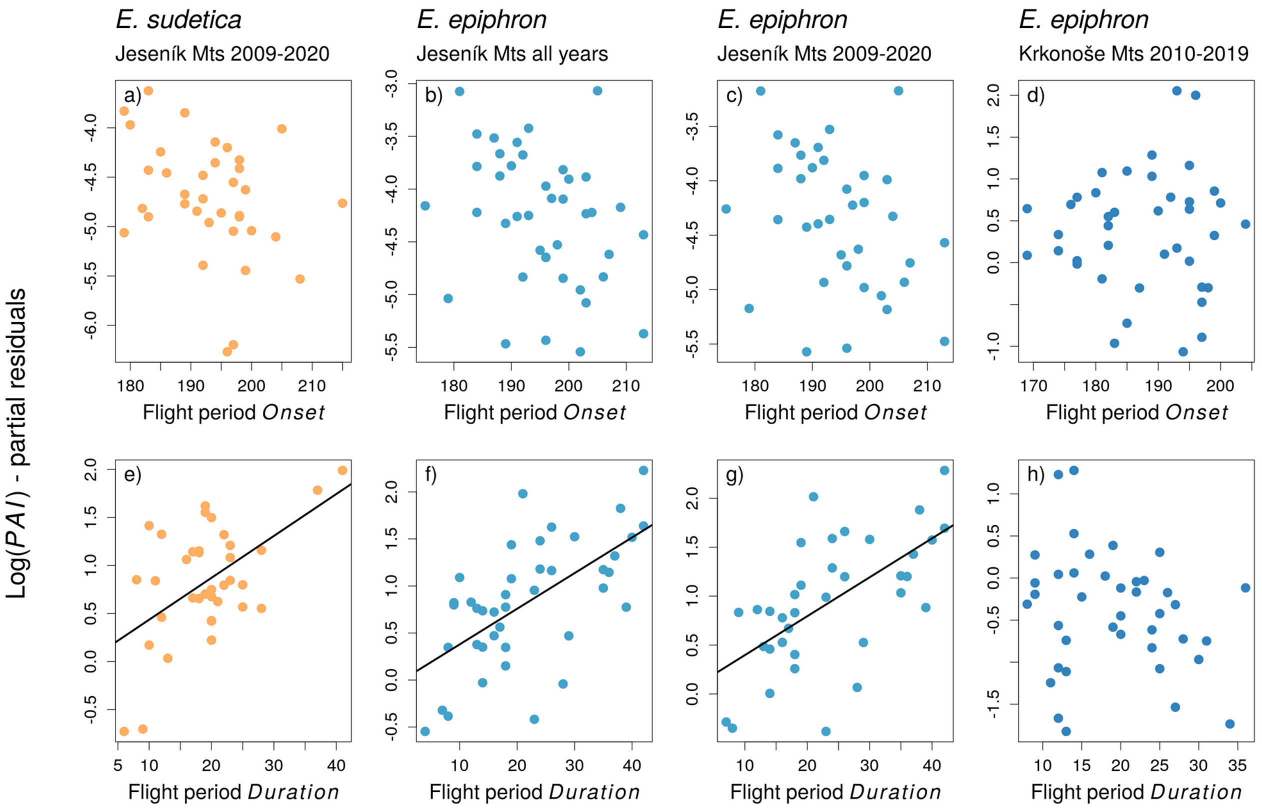

Fig. S17– The relationship between the phenology and abundance of *Erebia sudetica* and *E. epiphron*. A regression line is shown in cases when the regression coefficient was significantly different from zero (see Table 1 in the main text).

Temporal trends

We tested the dependence of the climate variables on the year separately for data from the Jeseník Mts (complete data including the 1990s and recent data from the period 2009–2020) and the Krkonoše Mts, where we used data from two weather stations – one at a lower altitude and another at a higher altitude. As expected, we detected several significant temporal trends in climate variables over the entire study period in the complete dataset from the Jeseník Mts, including measurements from the 1990s, but most of these trends were not significant over the short recent period of 2009–2020 (Table S2).

Figures S18 and S19 show the temporal trend, or the lack thereof, in the phenology of *Erebia sudetica* and *Erebia epiphron* in the two mountain ranges. We estimated the *Onset* and *Duration* of the flight period separately for each transect (see Methods in the main text). We tested the relationship between the *Onset* or *Duration* of the flight period (both log-transformed) and the year using generalised linear models (GLM) with a Gaussian error distribution. We used AIC to compare the fit of a model with transect identity and the year as predictors to a model with transect identity only. In most cases, the simpler model was better supported. Hence, there was no evidence of a shift in the *Onset* or *Duration* of the flight period in time over the duration of our study, with the exception of *E. epiphron* in the Jeseník Mts with data from all years, including the 1990s included in the analysis (see also Table 2 in the main text).

Table S2. Temporal trends in the climate variables tested using generalised linear models (GLM) with Gaussian error distribution. *ΔAIC* shows the difference in *AIC* of each listed model compared to a corresponding null model without the effect of the year; i.e. models with lower *AIC* compared to the null model have negative values of *ΔAIC*. We consider values of *ΔAIC* < -2 as **moderate** evidence of the effect of the year and *ΔAIC* < -6 as **strong** evidence of the effect of the year. The explanation of the variables is provided in the Methods in the main text. df = residual degrees of freedom. Empty cells (–): the climate variable not available for the mountain range and time period.

Model	Jeseník Mts All years				Jeseník Mts 2009–2020				Krkonoše Mts 2010–2019 (valley)				Krkonoše Mts 2010–2019 (summit)			
	ΔAIC	**Slope**	**SE**	**df**	***ΔAIC***	**Slope**	**SE**	**df**	***ΔAIC***	**Slope**	**SE**	**df**	***ΔAIC***	**Slope**	**SE**	**df**
P_{tot}Aut ~ Year	1.9	0.22	1.01	13	1.5	1.58	2.54	10	1.1	2.99	3.46	8	1.2	2.78	3.50	8
T_{avg}Aut ~ Year	**-8.3**	**0.12**	**0.03**	**13**	-1.3	0.14	0.08	10	-1.7	0.17	0.09	8	-0.7	0.20	0.13	8
T_{max}Aut ~ Year	-0.8	0.13	0.08	13	0.6	0.23	0.20	10	2.0	0.03	0.18	8	1.6	0.15	0.26	8
T_{min}Aut ~ Year	-1.8	0.08	0.04	13	1.9	-0.03	0.10	10	1.5	0.07	0.12	8	1.1	0.13	0.15	8
T_{ground}Aut ~ Year	–	–	–	–	2.0	0.01	0.12	10	–	–	–	–	–	–	–	–
P_{tot}Win ~ Year	1.2	0.38	0.45	13	**-4.2**	**2.63**	**1.01**	**10**	-0.8	2.90	1.80	8	-1.9	1.54	0.78	8
T_{avg}Win ~ Year	**-13.3**	**0.13**	**0.03**	**13**	-0.9	0.12	0.08	10	-0.8	0.17	0.11	8	0.5	0.12	0.11	8
T_{max}Win ~ Year	**-8.5**	**0.10**	**0.03**	**13**	0.0	0.10	0.07	10	2.0	-0.01	0.08	8	1.7	-0.03	0.07	8
T_{min}Win ~ Year	**-9.9**	**0.13**	**0.03**	**13**	0.4	0.10	0.09	10	**-2.1**	**0.27**	**0.13**	**8**	1.0	0.15	0.16	8
T_{ground}Win ~ Year	–	–	–	–	-0.9	0.20	0.12	10	–	–	–	–	–	–	–	–
$Snow_{days}$ ~ Year	–	–	–	–	1.2	-1.06	1.29	10	–	–	–	–	–	–	–	–
P_{tot}Spr~ Year	1.8	-0.74	1.61	13	1.6	-2.60	4.63	10	0.6	-4.40	4.06	8	1.2	-4.87	5.82	8
T_{avg}Spr ~ Year	**-7.3**	**0.10**	**0.03**	**13**	1.2	0.07	0.09	10	**-3.3**	**0.18**	**0.07**	**8**	-1.8	0.18	0.09	8
T_{max}Spr ~ Year	-0.2	0.06	0.04	13	0.9	0.11	0.12	10	1.6	0.08	0.15	8	1.4	0.09	0.13	8
T_{min}Spr ~ Year	**-5.0**	**0.11**	**0.04**	**13**	1.2	0.09	0.11	10	**-2.6**	**0.33**	**0.15**	**8**	**-2.5**	**0.27**	**0.13**	**8**
T_{ground}Spr ~ Year	–	–	–	–	0.7	0.11	0.10	10	–	–	–	–	–	–	–	–
P_{tot}Sum ~ Year	**-2.3**	**-4.60**	**2.22**	**13**	-0.2	-4.48	3.17	10	**-8.1**	**-13.21**	**3.54**	**8**	**-7.7**	**-16.40**	**4.54**	**8**
T_{avg}Sum ~ Year	**-9.5**	**0.13**	**0.03**	**13**	1.8	0.04	0.08	10	1.3	0.07	0.10	8	-0.1	0.14	0.10	8
T_{max}Sum ~ Year	**-7.1**	**0.18**	**0.06**	**13**	1.4	-0.09	0.13	10	1.7	-0.07	0.13	8	1.3	0.14	0.18	8
T_{min}Sum ~ Year	**-2.8**	**0.06**	**0.03**	**13**	1.9	0.02	0.06	10	2.0	0.00	0.10	8	1.5	0.07	0.11	8
T_{ground}Sum ~ Year	–	–	–	–	1.1	-0.06	0.07	10	–	–	–	–	–	–	–	–

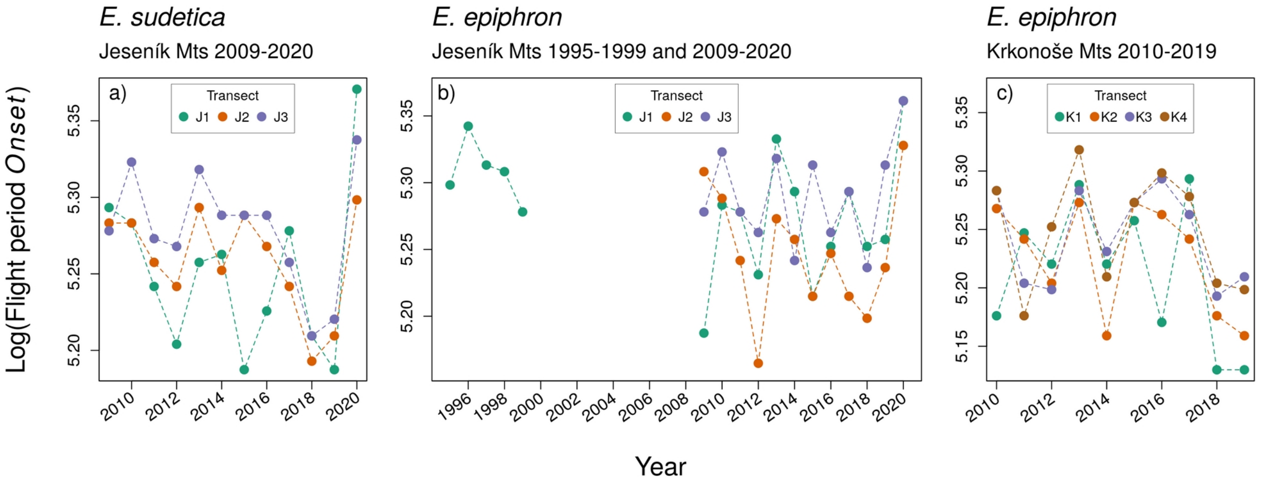

Fig. S18. The relationship between the *Onset* of the flight period, estimated separately for each transect, and the year of observation.

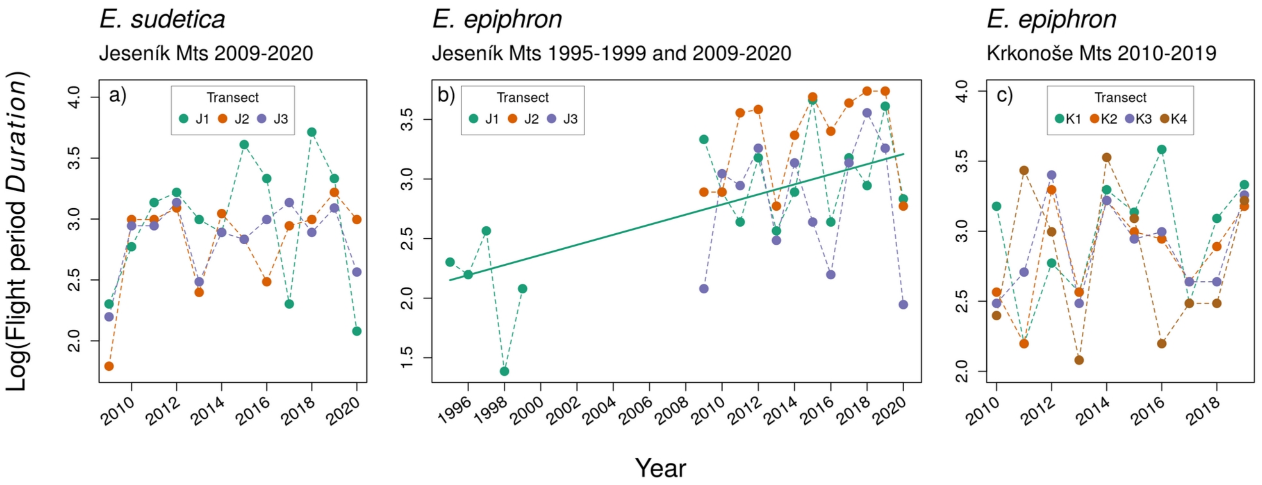

Fig. S19. The relationship between the *Duration* of the flight period, estimated separately for each transect, and the year of observation.

The effects of climate variables on abundance and phenology

Table S3 – The effect of the climate variables, each tested separately, on the *Onset* of the flight period (log-transformed). The climate variables were standardised to have zero mean and unit variance. *ΔAIC* = the difference of *AIC* of each listed model containing a given climate variable compared to the corresponding null model without the effect of the climate variable but also including the transect effect; i.e. log(*Onset*) ~ Transect. Hence, models with lower *AIC* compared to the null model have negative values of *ΔAIC*. Δ*AIC* < -6 was considered a **strong** and < -2 a **moderate** indication of the respective predictor's effect. Null df = residual degrees of freedom of the null model; all other models have df value lower by one. Empty cells (–): the predictor not available for the mountain range and time period.

**Model**	***E. sudetica* J 2009–2020 (Null df = 33)**			***E. epiphron* J_{all} 1995–1997 + 2009–2020 (Null df = 38)**			***E. epiphron* J_{rec} 2009–2020 (Null df = 33)**			***E. epiphron* K 2010–2019 (Null df = 36)**		
	ΔAIC	**Slope**	**SE**	***ΔAIC***	**Slope**	**SE**	***ΔAIC***	**Slope**	**SE**	***ΔAIC***	**Slope**	**SE**
Null model	0.0			0.0			0.0			0.0		
P_{tot}*Aut*	0.52	-0.01	0.01	1.23	-0.01	0.01	1.19	-0.01	0.01	**-3.84**	**-0.02**	**0.01**
T_{avg}*Aut*	1.87	0.00	0.01	1.94	0.00	0.01	1.74	0.00	0.01	1.60	0.01	0.01
T_{max}*Aut*	**-2.07**	**0.01**	**0.01**	0.20	0.01	0.01	-1.48	0.01	0.01	**-4.90**	**0.02**	**0.01**
T_{min}*Aut*	**-2.42**	**-0.01**	**0.01**	**-6.68**	**-0.02**	**0.01**	**-5.11**	**-0.02**	**0.01**	1.13	0.01	0.01
T_{ground}*Aut*	**-11.23**	**-0.02**	**0.01**	–	–	–	**-16.48**	**-0.02**	**0.01**	–	–	–
P_{tot}*Win*	-0.65	-0.01	0.01	1.98	0.00	0.01	2.00	0.00	0.01	-1.78	-0.03	0.02
T_{avg}*Win*	1.46	0.01	0.01	1.78	0.00	0.01	1.56	0.00	0.01	-0.09	-0.02	0.01
T_{max}*Win*	1.91	0.00	0.01	1.93	0.00	0.01	1.78	0.00	0.01	1.73	0.01	0.01
T_{min}*Win*	1.90	0.00	0.01	0.19	-0.01	0.01	1.93	0.00	0.01	2.00	0.00	0.01
T_{ground}*Win*	1.95	0.00	0.01	–	–	–	1.87	0.00	0.01	–	–	–
Snow$_{days}$	0.68	-0.08	0.07	–	–	–	1.44	0.05	0.07	–	–	–
P_{tot}*Spr*	**-11.15**	**0.02**	**0.01**	**-10.51**	**0.02**	**0.01**	**-10.41**	**0.02**	**0.01**	**-6.30**	**0.02**	**0.01**
T_{avg}*Spr*	**-24.54**	**-0.03**	**0.01**	**-10.72**	**-0.02**	**0.01**	**-8.35**	**-0.02**	**0.01**	**-3.02**	**-0.04**	**0.02**
T_{max}*Spr*	**-6.35**	**-0.02**	**0.01**	**-4.16**	**-0.02**	**0.01**	**-4.01**	**-0.02**	**0.01**	2.00	0.00	0.02
T_{min}*Spr*	**-8.12**	**-0.02**	**0.01**	-1.72	-0.01	0.01	-0.24	-0.01	0.01	**-2.52**	**-0.03**	**0.01**
T_{ground}*Spr*	0.21	-0.01	0.01	–	–	–	2.00	0.00	0.01	–	–	–

Table S4 – The effect of the climate variables, each tested separately, on the *Duration* of the flight period (log-transformed). The climate variables were standardised to have zero mean and unit variance. *ΔAIC* = the difference of *AIC* of each listed model compared to the corresponding null model without the effect of the climate variable but also including the transect effect; i.e. log(*Duration*) ~ Transect. Hence, models with lower *AIC* compared to the null model have negative values of *ΔAIC*. Δ*AIC* < -6 was considered as a **strong** and < -2 as a **moderate** indication of the respective predictor's effect. Null df = residual degrees of freedom of the null model; all other models have df value lower by one. Empty cells (–): the predictor not available for the mountain range and time period.

**Model**	***E. sudetica* J 2009–2020 (Null df = 33)**			***E. epiphron* J_{all} 1995–1997 + 2009–2020 (Null df = 38)**			***E. epiphron* J_{rec} 2009–2020 (Null df = 33)**			***E. epiphron* K 2010–2019 (Null df = 36)**		
	ΔAIC	**Slope**	**SE**	***ΔAIC***	**Slope**	**SE**	***ΔAIC***	**Slope**	**SE**	***ΔAIC***	**Slope**	**SE**
Null model	0.0			0.0			0.0			0.0		
P_{tot}*Aut*	1.08	-0.06	0.07	0.28	0.09	0.12	0.36	0.09	0.07	1.99	0.00	0.07
T_{avg}*Aut*	1.44	0.05	0.07	**-2.03**	**0.14**	**0.07**	0.38	0.09	0.07	-1.72	0.20	0.11
T_{max}*Aut*	-0.48	-0.10	0.07	-0.78	-0.12	0.17	**-3.70**	**-0.16**	**0.07**	1.92	-0.02	0.08
T_{min}*Aut*	0.55	0.08	0.07	**-11.34**	**0.24**	**0.06**	**-6.90**	**0.20**	**0.07**	1.99	-0.01	0.12
T_{ground}*Aut*	-1.40	0.12	0.07	–	–	–	**-4.50**	**0.24**	**0.06**	–	–	–
P_{tot}*Win*	0.25	0.09	0.07	-0.06	0.10	0.07	-0.45	0.11	0.07	1.96	0.03	0.13
T_{avg}*Win*	1.97	-0.01	0.07	0.71	0.08	0.07	1.92	-0.02	0.07	**-15.89**	**0.40**	**0.09**
T_{max}*Win*	-0.61	0.11	0.07	0.37	0.09	0.07	1.97	0.01	0.07	0.60	0.13	0.11
T_{min}*Win*	1.61	-0.04	0.07	0.40	0.09	0.07	1.98	-0.01	0.07	**-6.41**	**0.27**	**0.09**
T_{ground}*Win*	1.31	-0.06	0.07	–	–	–	2.00	0.00	0.07	–	–	–
Snow$_{days}$	0.68	-0.08	0.07	–	–	–	1.44	0.05	0.07	–	–	–
P_{tot}*Spr*	**-8.75**	**-0.21**	**0.06**	**-6.50**	**-0.19**	**0.07**	**-6.54**	**-0.19**	**0.07**	**-4.30**	**-0.16**	**0.06**
T_{avg}*Spr*	**-7.61**	**0.20**	**0.06**	**-11.92**	**0.25**	**0.06**	**-6.69**	**0.19**	**0.07**	1.57	-0.11	0.17
T_{max}*Spr*	**-3.20**	**0.15**	**0.07**	**-4.37**	**0.17**	**0.07**	**-2.96**	**0.15**	**0.07**	1.73	0.06	0.13
T_{min}*Spr*	**-3.87**	**0.16**	**0.07**	**-4.27**	**0.17**	**0.07**	-1.35	0.13	0.07	-0.12	0.18	0.13
T_{ground}*Spr*	1.07	0.06	0.07	–	–	–	1.93	0.02	0.07	–	–	–
P_{tot}*Sum*	1.84	-0.03	0.07	0.54	-0.09	0.07	1.25	-0.06	0.07	1.13	-0.06	0.07
T_{avg}*Sum*	1.36	0.05	0.07	**-6.00**	**0.19**	**0.07**	-0.82	0.12	0.07	1.98	0.02	0.15
T_{max}*Sum*	1.99	0.01	0.07	-0.90	0.12	0.07	1.89	0.02	0.07	**-5.44**	**-0.23**	**0.09**
T_{min}*Sum*	-1.03	-0.11	0.07	1.19	0.06	0.07	1.78	-0.03	0.07	1.93	0.04	0.18
T_{ground}*Sum*	1.21	-0.06	0.07	–	–	–	1.24	-0.06	0.07	–	–	–
